# Transcriptomic analysis reveals novel age-independent immunomodulatory proteins as a mode of cerebroprotection in P2X4R KO mice after ischemic stroke

**DOI:** 10.21203/rs.3.rs-2747807/v1

**Published:** 2023-03-31

**Authors:** Daylin Gamiotea-Turro, Chunxia C Cronin, Bruce T Liang, Rajkumar Verma

**Affiliations:** University of Connecticut Health Center; University of Connecticut Health Center; University of Connecticut Health Center; University of Connecticut Health Center

## Abstract

Identification of new potential drug target proteins and their plausible mechanisms for stroke treatment is critically needed. We previously showed that genetic deletion and short-term pharmacological inhibition of P2X4R, a purinergic receptor for adenosine triphosphate ATP, provides acute cerebroprotection. However, potential mechanisms remain unknown. Therefore, we employed RNA-seq technology to identify the gene expression profiles, pathway analysis, and qPCR validation of differentially expressed genes (DEGs). This analysis identified roles of DEGs in certain biological processes responsible for P2X4R-dependent cerebroprotection after stroke. We subjected both young and aged male and female global P2X4 KO and littermate WT mice to ischemic stroke. After 3 days, mice were sacrificed, total RNA was isolated using Trizol, and subjected to RNA-seq and Nanostring-mediated qPCR. DESeq2, Gene Ontology (GO), and Ingenuity Pathway Analysis (IPA) were used to identify mRNA transcript expression profiles and biological pathways. We found 2246 DEGs in P2X4R KO vs WT tissue after stroke. Out of these DEGs, 1920 gene were downregulated, and 325 genes were upregulated in KO. GO/IPA analysis of the top 300 DEGs suggests an enrichment of inflammation and extracellular matrix component genes. qPCR validation of the top 30 DEGs revealed downregulation of two common age-independent genes in P2X4R KO mice: Interleukin-6 (IL-6), an inflammatory cytokine, and Cytotoxic T Lymphocyte-Associated Protein 2 alpha (Ctla2a), an immunosuppressive factor. These data suggest that P2X4R-mediated cerebroprotection after stroke is initiated by attenuation of immune modulatory pathways in both young and aged mice of both sexes.

## 1. Introduction

Ischemic stroke is the leading cause of disability in the United States. It is a vascular accident that occurs, often without warning, when there is a blockage of cerebral blood flow, and results in damage to the surrounding brain tissue [1]. Despite recent advances, interventions to reduce damage and increase recovery after stroke are limited. Therapies, such as physical, occupational, and speech, are the only currently viable options available for post-stroke recovery [2]. This prompted us to explore pathophysiological events and mechanisms of injury to identify novel drug targets and their downstream pathways. Acute ischemic stroke triggers a series of events: rapid activation of resident microglia, release of chemokines and cytokines in the circulation, mobilization of immune cells from bone marrow, and the infiltration of circulating immune cells in the brain, primarily to phagocytose dead cells [3]. However, excessive infiltration of these immune cells into the ischemic region creates unwanted inflammation during the acute stroke phase by releasing inflammatory cytokines which leads to secondary damage after stroke, thus delaying recovery [4]. Among in filtrating cells, migration of peripheral myeloid cells (i.e., monocytes and neutrophils) to the CNS and their temporal contribution to ischemic injury is a well-known immune response. Peripheral (e.g., monocytes/macrophages) and CNS-resident (microglia) myeloid cells contribute to both acute injury and functional recovery after stroke [5]. Despite the crucial participation of myeloid cells in stroke outcomes, approaches to regulate myeloid cell response remain unsuccessful due to their complex function including both cerebroprotective and damaging roles in stroke.

The effects of aging on the immune system occur at multiple levels, including reductions in lymphoid and increases in myeloid progenitor cells by hematopoietic stem cells [6]. Despite increases in their total systemic number, myeloid cells from aged mice are not as robust as their young counterparts and show diminished phagocytosis and reduced production of cerebroprotective secretory molecules; however, aged immune cells show increased secretion of inflammatory mediators [7]. We previously showed that aged mice exhibit marked differences in the composition of circulating and infiltrating leukocyte recruitment after stroke [7]. Aged microglia also show increased sensitivity to ATP [8]. Therefore, we hypothesize that P2X4R activation in aged mice with ischemic stroke may show more pronounced neuroinflammatory cytokine/chemokine gene expression as compared to young mice. We also hypothesize that there will be some age-independent differentially expressed genes (DEGs) contributing to cerebroprotective or cerebrodetrimental effects. We will test this hypothesis by directly comparing P2X4R response in young and aged mice after stroke.

Our previous work revealed that the acute overactivation of P2X4, a purinergic receptor for ATP, exacerbated post-ischemic inflammation and hampered recovery [9]. Using short-term pharmacological blockade and genetic deletion, we showed that P2X4R can be a potential drug target for the treatment of acute ischemic stroke [10]. In the CNS, besides its acute neuroinflammatory role, P2X4 also plays a significant role in modulating synaptic transmission and communication between neurons and neighboring glial cells [11]. We and others have shown that myeloid cells express the highest amount of P2X4R and can modulate neuroinflammation mediated by chemokines, cytokines, and secondary activation of other immune cells such as T cells [10, 12, 13]. However, its molecular mechanism remains unknown. Here we used bulk RNAseq analysis using ischemic brain tissue from P2X4R KO and WT mice to identify common pathways and molecular mediators of damage and recovery. Transcriptomic analysis, which involves measuring the expression levels of thousands of genes simultaneously, provides a comprehensive view of the molecular changes that occur after ischemic stroke and allows for the identification of DEGs that may be involved in the pathophysiology of the disease [14].

The use of transcriptomic analysis in this study can help identify the specific genes and pathways that are affected by the knockout of P2X4R and can shed light on the potential mechanisms by which P2X4R may be involved in ischemic stroke. This information can then be used to develop targeted therapies that specifically modulate the expression or activity of these genes and pathways.

## 2. Methods

### 2.1. Animals and diets

We used both young (2–3-month-old) and aged (16–18-month old) Global P2X4R knockout (KO) and littermate control (WT) mice of both sexes, generated in-house at the UConn Health animal facility. Mice were fed a standard chow diet and water ad libitum. Standard housing conditions were maintained at a controlled temperature with a 12-h light/dark cycle. All experiments were approved by the Institutional Animal Care and Use Committee of University of Connecticut Health and conducted in accordance with the U.S. National Institutes of Health Guidelines for the Care and Use of Laboratory Animals.

#### Experimental design and stroke surgery:

A total of 22 young and 26 aged mice (containing both males and females) were used in this study. Out of 48 mice, 32 mice (were subjected to a transient right middle cerebral artery occlusion (MCAo) for 60 min followed by 3 days of reperfusion as described previously [9, 10], while 10 mice (4 young and 6 aged) were subjected to sham surgery and the remaining 6 naïve young mice were used for bone marrow cell isolation. For MCAo surgery, a midline ventral neck incision was made to expose the right common carotid artery which was followed by isolation of both the external and internal carotid artery. A unilateral right MCAo was achieved by inserting a 6.0 silicone rubber-coated mono lament (size 602145/602245/602345; Doccol Corporation, Sharon, MA) about 10–11 mm away from the bifurcation point of the internal carotid artery through an external carotid artery stump. After 60 mins of occlusion, mono lament was removed to perfuse the brain tissue. Rectal temperatures were monitored and maintained at 37 ± 0.5°C with the help of a heating pad. We used laser Doppler flowmetry (DRT 4/Moor Instruments Ltd, Devon, UK) to measure cerebral blood flow and to confirm occlusion (reduction to 15% of baseline cerebral blood flow). Four aged mice were died after stroke so final data includes total 44 mice.

### 2.2. Brain tissue isolation and total RNA extraction

Three days after MCAo, all the mice were sacrificed and perilesional prefrontal cortex tissue was isolated from the ischemic hemisphere to extract total RNA using Trizol. We used 500 ng and 200 ng RNA from each sample for RNA-seq sample preparations (Illumina Hi Seq, Yale Center for Genome Analysis, New Haven, CT) and nano string platform (IDDRC molecular genetics core at BOSTON CHILDREN’S HOSPITAL, Boston, MA), respectively.

### 2.3. Bone marrow derived monocyte (BMDM) isolation:

After sacrifice, P2X4R KO and littermate WT mice (8–10 weeks old) were sacrificed and bones (femur and tibia) were harvested after careful dissection in aseptic conditions. The harvested bone was cut from both ends and the internal lumen was flushed with RPMI media into a collecting tube. Following RBC lysis, single cell suspension was poured into a culture dish to grow for 10–14 days. Bone marrow-derived cells were fed with GM-CSF (10 ng/ml) to differentiate into macrophages. Once macrophage cultures were established, a comparative study was conducted between macrophages of P2X4R KO and WT groups under defined conditions. At the end of the experiment, the harvested cells were used for total RNA isolation using Trizol.

### 2.4. RNA-seq analysis

#### 2.4.1. Quality Control

Total RNA quality was determined by estimating the A260/A280 and A260/A230 ratios by nanodrop. RNA integrity was determined by running an Agilent Bioanalyzer gel, which measures the ratio of the ribosomal peaks.

#### 2.4.2 RNA-Seq Library Prep

mRNA was purified from approximately 500 ng of total RNA with oligo-dT beads and sheared by incubation at 94°C. Following first-strand synthesis with random primers, second strand synthesis was performed with dUTP for generating strand-specific sequencing libraries. The cDNA library was then end-repaired, A-tailed, adapters were ligated, and second-strand digestion was performed by Uracil-DNA-Glycosylase. Indexed libraries that met appropriate cut-offs for both were quantified by qRT-PCR using a commercially available kit (KAPA Biosystems) and insert size distribution determined with the LabChip GX or Agilent Bioanalyzer. Samples with a yield of ≥ 0.5 ng/μl were used for sequencing.

#### 2.4.3 Flow Cell Preparation and Sequencing

Sample concentrations were normalized to 10 nM and loaded onto Illumina HiSeq4000 flow cells at a concentration that yields 300 million passing filter clusters per lane. Samples were sequenced using 100 bp paired-ends sequencing according to Illumina protocols. The 8 bp index was read during an additional sequencing read that automatically follows the completion of read 1. Data generated during sequencing runs were simultaneously transferred to the YCGA high-performance computing cluster. A positive control (prepared bacteriophage Phi × library) provided by Illumina was spiked into every lane at a concentration of 0.3% to monitor sequencing quality in real time.

#### 2.4.4 Primary RNA-Seq Analysis and Storage

Signal intensities were converted to individual base calls during a run using the system’s Real Time Analysis (RTA) software. Base calls were transferred from the machine’s dedicated personal computer to the High-Performance Computing cluster at Yale Center for Research Computing (New Haven, CT) via a 1 Gigabit network mount for downstream analysis.

#### 2.4.5 Secondary RNA-Seq Data Analysis

Our analysis was carried out as follows. The reads were trimmed for quality using custom scripts. Minimum length accepted was 45 bases. The trimmed reads were then aligned to the mm10 reference genome using gencode annotation (Frankish et al., 2019) using HISAT2 for alignment, and StringTie for transcript abundance estimation (Kim et al., 2015). The generated counts were processed with DESeq2 (Love et al., 2014) in R to determine significantly expressed genes. Internally, the DESEq2 uses Wald’s test for each gene to determine if the log fold change is statistically significant. Wald test itself is a variation of χ^2^ test (This is one reason why one needs to use the multiple hypothesis testing correction to determine truly statistically significant genes). For the current analysis, we used adjusted p-value < 0.05 (effectively accepting 5% false discovery rate). The adjustment applied to the p-value is one of the mildest corrections.

### 2.5. Differential expression gene analysis (DEG analysis)

Fragments per kilobase of exon model per million mapped reads (FPKM) is the most commonly used method for measuring gene expression levels (Mortazavi et al., 2008). The gene expression level of each sample was analyzed by HTSeq software (Princeton University, USA), using union as the counting model. We used a cutoff value of FPKM > 1 to define the gene expression. DEG analysis was performed using DESeq (Anders and Huber, 2010) (Bioconductor, USA). Genes with differential expression were screened and hierarchical clustering analysis was performed.

### 2.6. Gene Ontology (GO) analysis

Gene ontology analysis was performed on the sets of top 10% up and down regulated genes in P2X4R KO mice from RNAseq data after stroke. Using the GO database (http://www.geneontology.org/), biological processes with an FDR threshold of ≤ 0.05 were filtered and biological process-related categories were selected and grouped by hierarchy [15].

### 2.7. Pathway and network analysis by Ingenuity Pathway Analysis (IPA).

Similar to GO analysis, a list of DEG (top 300) containing gene identifiers and corresponding expression values was uploaded into the IPA software (Qiagen) [16]. The “core analysis” function included in the software was used to interpret the differentially expressed data, which included biological processes, canonical pathways, upstream transcriptional regulators, and gene networks. Each gene identifier was mapped to its corresponding gene object in the Ingenuity Pathway Knowledge Base (IPKB).

### 2.8 QPCR validation of top DEGs by NanoString Technology:

A total of 200 ng of RNA per sample was sent to Molecular Genetics Core Facility Boston Children’s Hospital, Boston, MA for gene expression profiling using the nCounter platform and custom Codeset of the 30 top DEGs and housekeeping genes (Table 1). Data was normalized as per manufacturer’s instructions by using the nSolver Software (NanoString Technologies) and used for differential expression as in [10].

## 3. Results

We performed RNA-sequencing (RNA-seq) using total RNA isolated from perilesional ipsilateral cortex of young P2X4R KO and WT mice. After quality filtering and normalizing the raw sequencing data, we identified DEGs based on the following criteria: fold change > 2 or < 0.5, and false discovery rate (FDR) < 0.05. To understand the molecular and cellular impacts of these DEGs, we analyzed the top 300 genes (consisting of both up- and down-regulated genes) among KO vs. WT mice after stroke using GO and IPA analysis. Among the top gene candidates, we validated about 30 biologically relevant genes using nanostring technology.

### 3.1. Quality control of RNA seq data:

We used several parameters like nReads, p.Unique, p.Aln, n.Unique, p.Coding.UTR, p.Intronic, p.Ribo, p.Intergenic, etc., as given in Table 1.

### 3.2. DEGs identification and enrichment

Compared with WT control, 2245 genes were differentially expressed in the P2X4R KO group, with FDR (p adjusted) value < 0.05, including 325 up-regulated genes and 1920 down-regulated genes (Supplementary File 1). The top 300 DEGs were then subjected to GO and IPA analysis for gene enrichment and network analysis. Other comparison between Sham P2X4R KO and WT as well as Sham and stroke are given in supplementary data (Supplementary File 1)

### 3.3. GO analysis

Using the top 300 DEGs, we identified 17 total significantly enriched GO terms (Table 2) with 11 related to biological process and 6 related to molecular function. The top hit in the biological process and molecular function category were related to extracellular matrix, cellular origination, angiogenesis, and inflammatory response function (Fig. 1).

### 3.4. Networks analysis of candidate genes by IPA

**Figure 2:**
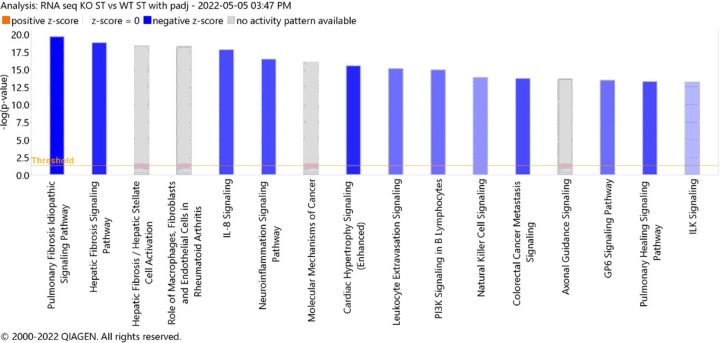
Histogram of the top sixteen canonical pathways (p, 0.05) for young P2X4R KO vs WT stroke group. Ingenuity Pathway Analysis (IPA) of top 16 canonical pathways for each genotype. The most statistically significant canonical pathways identified in the brain tissue are listed according to their p value (−log; orange line). Blue bars: negative z-score; orange bars: positive z-score; gray bars: no activity pattern available. The y–axis represent the −log (p-value).

We subjected DEGs regulating the three functions to IPA gene network analysis. IPA explores the set of input genes to generate networks by using Ingenuity Pathways Knowledge Base for interactions between genes. We used two metrics to identify the most important downstream effects of these 300 DEGs: activation z-score and p-value (p > 12.5) (Fig. 2). A negative z-score (Blue bars) indicates pathway inhibition in P2X4R KO vs. WT. The p-value, calculated with the Fischer’s exact test, indicates the likelihood that the association between a set of genes in our dataset and a biological function is significant. Analysis of the top 16 pathways identified that P2X4R regulates 12 main functions connected to several molecular pathways (Fig. 1). Among these, neuroinflammatory signaling pathways and immune cell activation were prominent.

### 3.5 qPCR validation of the top 30 biologically relevant genes.

**Figure 3:**
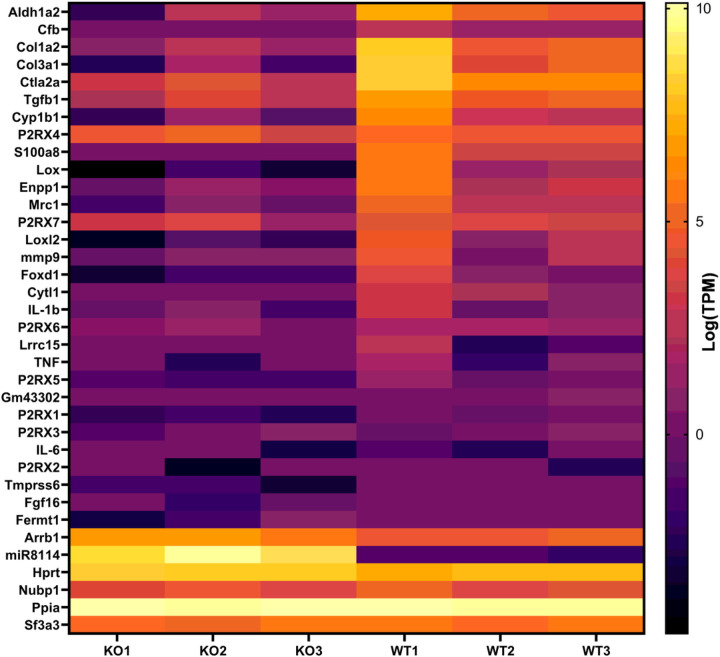
Heat map of top 30 DEGs hierarchical clustering analysis. The right 3 columns represent WT controls, and the left 3 columns represent P2X4R KO mice. Each row represents a single gene. The color change from yellow to black represents log (TPM) value ranging from high to low. This heat map represents expression data of top 30 immune related genes validated by nano string panel using mouse brain tissue of young, stroked mice (n=3/genotype).

**Figure 4:**
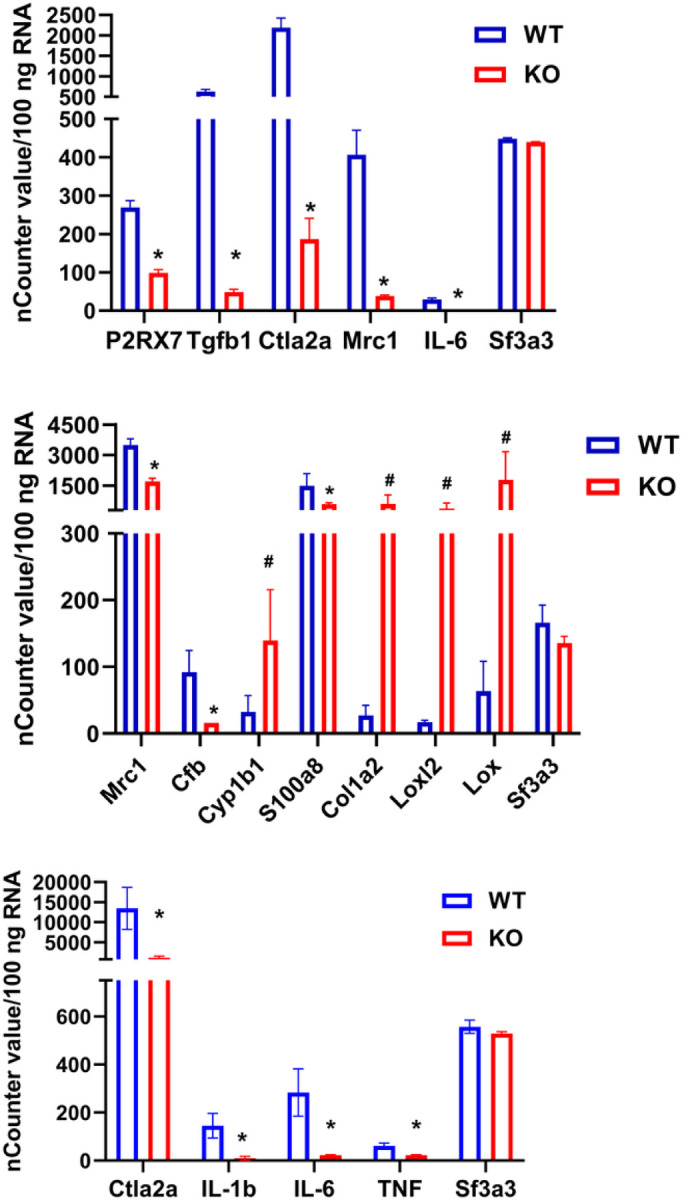
qPCR validation of top 30 DEGs related to inflammatory genes. A) Young P2X4 KO & WT mice (n=3/genotype), B) BMDMs (n=3/genotype), C) Aged P2X4 KO & WT mice (n=8/genotype), Ctla2a and Il-6 mRNA was downregulated in KO mice of both age group after stroke *p<0.05 vs WT (paired T test),. Data are mean ± S.D.

Hierarchical clustering analysis of the top 30 biologically relevant DEGs showed (Fig. 3) that most differentially regulated genes were related to extracellular matrix, cell-cell adhesion and immunomodulatory pathways. Further, we identified several significantly modulated inflammatory cytokines or chemokines in young brain tissue (Fig. 4a) and BMDMs (Fig. 4b) and aged brain tissue (Fig. 4c). We identified many novel downstream targets of P2X4R in the transient MCAo model of ischemic stroke.

## 4. Discussion

Our previous study demonstrated that Global P2X4R KO protects mice from further damage after ischemic stroke, yet the underlying molecular networks associated with P2X4R in the neuro-inflammatory response to cortical injury due to ischemic stroke remain unexplored [9]. We used next-generation RNAseq to analyze gene expression profiles generated from P2X4R KO and littermate WT mice to identify potential biological and molecular function pathways and related signaling networks in the absence of P2X4R during acute neuro-inflammation after ischemic stroke injury using transient MCAo mouse model. Here, we have identified many up- and down-regulated genes, revealing that the absence of P2X4R has widespread effects after ischemic stroke.

Gene ontology (GO) enrichment analysis of DEGs suggest many biological processes such as cellular integrity, organization, positive regulation of angiogenesis, and inflammatory response modulation are among the top biological processes which are affected by the absence of P2X4R. The dependence of these biological processes on P2X4R was further supported by molecular function pathways by GO analysis and canonical pathways by IPA. These two independent analysis platforms suggest that immune cell activation/neuroinflammation and leukocyte extravasation pathways/function are dominantly altered by the absence of P2X4R. We and others have previously shown that P2X4R activation exacerbates inflammation and acute ischemic injury [3, 510]. We previously showed that short-term blockade of P2X4R reduces acute ischemic injury by reducing neuroinflammation and by reducing leukocyte infiltration into the brain after stroke [10]. These data also suggest that P2X4R plays a significant role in blood-brain barrier (BBB) integrity, either by activation of P2X4R on myeloid cells or its interaction with endothelial cells. However, it is not yet clear if these effects on BBB permeability are mediated by myeloid or endothelial P2X4R activation [10, 11]. Our qPCR data with BMDM cells suggest that the absence of P2X4R increases transcripts like COL1A2, LOX, and LOXL2. These genes are involved in rebuilding of ECM by cross-linking collagen and elastin fibers, which can improve the brain’s structural integrity and support its recovery following a stroke. It is well established that ECM integrity is compromised after stroke [17]. These findings support the notion that myeloid P2X4R activation might play a major role in ECM degradation and cell to cell adhesion, thus may affect BBB integrity.

Our qPCR data validated data from young, stroked mice showing that the absence of P2X4R reduced several inflammatory transcripts like P2X7R, Tgfb1, and MRC1. Among them, MRC1, which is a member of the C-type lectin (CLEC) family of mannose receptor, has diverse roles and can bind and internalize a variety of endogenous and pathogen-associated ligands [18]. Because of these properties, its role in endocytosis as well as antigen processing and presentation has been studied intensively and is consistent with the role of P2X4R in endocytosis [19]. Our data suggest that the endocytosis roles of P2X4R might be mediated by MRC1. Besides endocytosis, it can also directly influence the activation of various immune cells by its expression during ischemic stroke [20]. Both ischemic brain tissue in young mice and BMDM data show significant decrease in MRC1 transcript in P2X4R KO group, indicating that cerebroprotective effects of P2X4R blockade might be mediated by MRC1-mediated immune cell activation. MRC1 also has a regulatory effect on the induction of immune responses which are distinct from antigen uptake and presentation, specifically it regulates T-cell activation [21]. This regulatory effect of the MRC1 was mediated by a direct interaction with CD45 on the T cell, inhibiting its phosphatase activity, which resulted in up-regulation of cytotoxic T-lymphocyte-associated protein. Given that the mannose receptor plays an important role in phagocytosis and clearance of cellular debris [22], it will be worthwhile to study how P2X4R blockade affects phagocytic uptake and T-cell activation after ischemic stroke. Our data suggest that MRC1-P2X4R may play important diverse roles during acute ischemic injury. Interestingly, our data found an age-independent decrease in expression of transcript Cytotoxic T-lymphocyte antigen-2 alpha (CTLA-2a) in P2X4R KO mice after stroke. This finding suggests that the effects MRC1 on T cells might be mediated by CTLA-2a in a P2X4R-dependent manner persisting in aging.

CTLA-2a is a cysteine proteinase inhibitor which was originally discovered in mouse-activated T cells and mast cells. Previously it has been shown that T cell activation is detrimental during acute ischemia, and lymphocyte-deficient mice are protected in models of focal ischemia as discussed in detail in [3]. The cytotoxic activity of T cells may be related to innate functions of T cells. Further, P2X4R activation increases T cell activation and their migration to injured tissue [13]. This evidence suggests that loss/blockade of P2X4R in the brain reduces CTLA-2a expression on T cells to inhibit their activation and migration to ischemic tissue, which is consistent with IP analysis data. CTLA2a was reduced both in young and aged mice after stroke and stroke mainly occurs in aging population. This observation suggests that CTLA-2a may be a downstream target of P2X4R and can be a potential therapeutic target to treat ischemic stroke. Similar to CTLA-2a, we also found reduced expression of IL-6 after stroke in both young and aged P2X4R KO mice. Although the pro-inflammatory role of IL-6 is well-defined after acute stroke injury, reduced IL-6 levels may indicate cerebroprotective effects of P2X4R KO during early stroke injury timepoints. Although the exact mechanism of how P2X4R activates IL-6 is not clear, we and others have shown that pro-inflammatory effects of P2X4R activation might indirectly contribute in stroke injury [23].

Taken together, our RNAseq data support the hypothesis that P2X4R modulates BBB integrity, inflammatory response, and immune cell activation and infiltration. This data also identifies two novel potential downstream targets of P2X4R during ischemic stroke: MRC1 and CTLA-2a. Data from this work support not only a key role for P2X4R in modulating the complex networks of cell death and the immune response in myeloid cells after ischemic stroke but also suggest a new role in T cell activation and a potential mechanism for this T-cell activation.

## Figures and Tables

**Figure 1:** Gene ontology (GO) enrichment analysis for top 300 DEGs (A) Biological Process and (B) Molecular Function. Each pie displays the distribution of DEGs according to biological process and molecular function. The top 6 significantly enriched biological processes and molecular functions are shown along with the p-value after FDR and number inside the Pie suggest percent DEGs contributing those pathways. The pathways related to immunomodulation (calcium binding, immune receptor activity and angiogenesis) are marked in bold in each case. The GO enrichment analysis was performed using the PANTHER Classification System.

**Table 1:** RNA Seq Metrics

Sample	nReads	p.Unique	p.Aln	n.Unique	p.Coding.UTR	p.Intronic	p.Ribo	p.Intergenic
WT1	37281768	67.65%	86.72%	25218490	76.93%	11.89%	2.22%	8.99%
WT2	52533142	66.41%	86.83%	34881254	67.07%	16.46%	2.38%	14.11%
WT3	41843688	73.13%	90.13%	30595240	76.50%	11.71%	1.38%	10.43%
KO1	49133748	65.67%	85.76%	32259060	68.85%	15.61%	3.37%	12.21%
KO2	48974522	70.05%	87.96%	34301356	71.52%	16.01%	2.54%	9.98%
KO3	50459042	67.94%	87.70%	34277640	72.12%	13.33%	1.83%	12.74%

**Table 2:** GO analysis pathways

Name of the process	Description and GO accession	DEG item	Fold enrichment	FDR	total read
Biological process	extracellular matrix organization (GO:0030198)	24	7.72	7.97E-10	291
Biological process	regulation of cellular component movement (GO:0051270)	40	3.16	5.29E-07	291
Biological process	blood vessel morphogenesis (GO:0048514)	24	4.83	9.09E-07	291
Biological process	positive regulation of angiogenesis (GO:0045766)	15	7.58	3.97E-06	291
Biological process	inflammatory response (GO:0006954)	23	4.1	1.48E-05	291
Biological process	cell adhesion (GO:0007155)	31	3.17	1.51E-05	291
Biological process	RNA processing (GO:0006396)	0	< 0.01	2.20E-02	291
Biological process	RNA metabolic process (GO:0016070)	3	0.21	4.38E-02	291
Biological process	sensory perception of smell (GO:0007608)	1	0.08	1.10E-02	291
Biological process	sensory perception of chemical stimulus (GO:0007606)	1	0.07	4.10E-03	291
Biological process	sensory perception (GO:0007600)	5	0.27	3.08E-02	291
Molecular function	extracellular matrix structural constituent (GO:0005201)	16	9.54	2.57E-07	291
Molecular function	calcium ion binding (GO:0005509)	27	3.81	1.37E-05	291
Molecular function	integrin binding (GO:0005178)	12	6.97	5.61E-04	291
Molecular function	glycosaminoglycan binding (GO:0005539)	14	5.35	9.12E-04	291
Molecular function	collagen binding (GO:0005518)	8	8.94	4.85E-03	291
Molecular function	immune receptor activity (GO:0140375)	10	6.62	4.94E-03	291

## Data Availability

Some of the raw data of RNAseq is provided in supplementary file and other data will be available on demand from corresponding author due process mandated by UConn Health from an authorized entity.
